# From persistent hypercalcemia to parathyroid carcinoma: a case report of acute urinary retention and the diagnostic role of Ki-67

**DOI:** 10.3389/fmed.2026.1755015

**Published:** 2026-05-04

**Authors:** Chang Deng, Chun Huang, Xinliang Su

**Affiliations:** 1 Chongqing University of Technology, The Central Hospital Affiliated to Chongqing University of Technology (The Seventh People’s Hospital of Chongqing), Chongqing, China; 2 Department of Breast and Thyroid Surgery, The First Affiliated Hospital of Chongqing Medical University, Chongqing, China

**Keywords:** acute urinary retention, case report, hypercalcemia, Ki-67, parathyroid carcinoma

## Abstract

**Background:**

Parathyroid carcinoma (PC) is a rare and often misdiagnosed endocrine malignancy that can present with diverse clinical manifestations, complicating its detection and management.

**Case presentation:**

This case report presents a 59-year-old male diagnosed with parathyroid carcinoma (PC), initially misidentified as benign parathyroid hyperplasia and adenoma. Over the course of 4 years, the patient underwent three surgical interventions. Final surgery confirmed the definitive diagnosis of PC, which revealed vascular invasion. The patient exhibited unusual clinical manifestations, notably severe hypercalcemia leading to acute urinary retention (AUR), which posed a diagnostic challenge. Pathological evaluation indicated a Ki-67 proliferation index of 8%, raising concerns about malignancy. This case highlights the need for multidisciplinary collaboration in the diagnostic process, recognizing atypical symptoms and carefully interpreting histopathological markers in suspected PC cases.

**Conclusion:**

Persistent hyperparathyroidism, elevated Ki-67 levels, and unexplained lower urinary tract symptoms (LUTS) should prompt re-evaluation for malignancy, highlighting the rarity yet clinical significance of hypercalcemia-induced AUR as a presenting symptom of underlying parathyroid pathology.

## Introduction

Parathyroid carcinoma (PC) is an exceptionally rare endocrine malignancy that accounts for less than 1% of all cases of primary hyperparathyroidism (1, 2). This malignancy is characterized by excessive secretion of parathyroid hormone (PTH), which leads to severe hypercalcemia and associated complications. The clinical presentation of PC often overlaps with that of benign parathyroid conditions, such as adenomas and hyperplasia, complicating the diagnostic process. The symptoms of hyperparathyroidism may include fatigue, weakness, nephrolithiasis, and gastrointestinal disturbances (1, 3, 4). However, in some cases, patients may present with atypical manifestations such as acute urinary retention (AUR), which can be attributed to severe hypercalcemia. The diagnostic criteria for PC remain challenging, as definitive histopathological features, such as capsular or vascular invasion, may not be apparent in the early stages or could be misinterpreted as benign during intraoperative assessments (5, 6). AUR as the initial presenting symptom of hypercalcemia is exceptionally rare. A comprehensive literature search revealed that while hypercalcemia is a well-recognized cause of polyuria, polydipsia, and nephrolithiasis in the context of primary hyperparathyroidism, AUR has been documented as the sentinel event leading to a diagnosis of parathyroid carcinoma in only isolated case reports worldwide (7). The majority of published case series on parathyroid carcinoma describe typical manifestations such as bone pain, nephrolithiasis, fatigue, and gastrointestinal symptoms; urinary retention is not listed among the common presenting features in major reviews (8–10). This case represents an unusual presentation wherein severe hypercalcemia precipitated AUR in a patient with underlying benign prostatic hyperplasia, highlighting a diagnostic pitfall that warrants heightened clinical awareness.

This case report describes the clinical course of a 59-year-old male patient who presented with an uncommon manifestation of parathyroid carcinoma (PC), initially misdiagnosed as benign parathyroid hyperplasia and adenoma. Over 4 years, the patient underwent three surgical procedures before a definitive diagnosis of PC was established, underscoring the diagnostic challenges associated with atypical presentations. This case emphasizes the importance of multidisciplinary collaboration in complex diagnostic scenarios. The rare clinical manifestation of acute urinary retention (AUR) induced by severe hypercalcemia, along with a Ki-67 proliferation index of 8%, highlights the need for careful interpretation of histopathological markers and heightened clinical suspicion in patients presenting with unexplained urinary symptoms in conjunction with hypercalcemia (11, 12).

## Case presentation

### Patient information (as visualized in the disease timeline)

**FIGURE 1:**
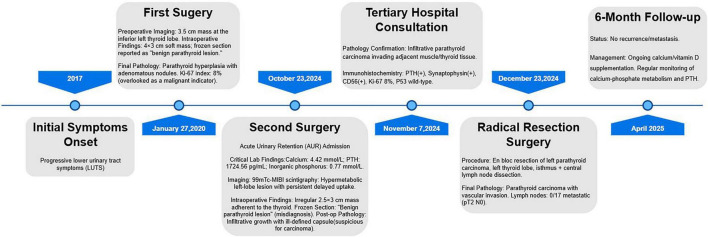
Timeline of parathyroid carcinoma progression.

A 59-year-old male presented with progressive urinary difficulty for 7 years and acute urinary retention for 1 day (Figure 1). He reported increased nocturia (7–8 times per night, 50 mL per void), urinary frequency, urgency, weak stream, hesitancy, stream bifurcation, terminal dribbling, and incomplete bladder emptying. One day before admission, he developed a sudden inability to urinate accompanied by severe lower abdominal pain, urinary dribbling, palpitations, and shortness of breath, but no nausea, vomiting, cough, sputum, chills, or fever. In the emergency department, an indwelling catheter was inserted to drain 1,000 mL of pale-yellow urine to relieve abdominal pain. He was admitted to the urology department with a diagnosis of “benign prostatic hyperplasia (BPH) and AUR.”

The patient had a known history of benign prostatic hyperplasia managed conservatively without prior episodes of acute urinary retention. The acute onset of AUR occurred in the context of severe hypercalcemia (4.42 mmol/L), and prompt catheterization relieved symptoms. No urological intervention for BPH was required, supporting hypercalcemia as the precipitating factor.

The patient had undergone surgery for primary hyperparathyroidism at another hospital on 27 January 2020. Imaging showed a 3.5 cm mass in the inferior portion of the left thyroid lobe, suggestive of hyperfunctioning parathyroid tissue. Surgery revealed a 4 × 3 cm soft mass, and an intraoperative frozen section identified a “benign parathyroid lesion.” Post-operative pathology revealed parathyroid tissue hyperplasia, adenomatous nodules, and focal fibrous tissue proliferation. Immunohistochemistry results were as follows: PTH (+), CgA focal (+), Ki-67 8% (+), calcitonin (−), and TG (−), with incomplete capsule formation.

Upon admission, the patient’s calcium level was 4.42 mmol/L (normal range: 2.11–2.50 mmol/L) and inorganic phosphorus 0.77 mmol/L (normal range: 0.85–1.51 mmol/L). Endocrinology consultation recommended diuretics, salmon calcitonin, aggressive fluid resuscitation, and close monitoring of electrolytes and vital signs. If calcium remained elevated, nephrology consultation for possible dialysis was advised. A parathyroid tumor was suspected, and the patient was referred for further evaluation and treatment.

### Physical examination

The neck was symmetrical to the trachea at the midline. A chronic surgical scar is observed in the anterior neck. A firm, non-tender, mobile mass measuring approximately 3.0 cm × 2.5 cm × 2.0 cm was palpated in the left thyroid lobe, with unclear boundaries. The right thyroid lobe appeared normal, and no enlarged lymph nodes were observed in the neck. Carotid pulsations were normal with no jugular venous distention, and hepatjugular reflux (HJR) was negative.

### Laboratory investigations

Parathyroid hormone: 1724.56 pg/ml (normal range: 11–81 pg/ml).

1. Calcium: 3.36 mmol/L (normal range: 2.11–2.50 mmol/L).
2. Inorganic phosphorus: 0.54 mmol/L (normal range: 0.85–1.51 mmol/L).

Thyroid function test results were within normal limits.

All laboratory readings (pre- and post-operative) are presented in Table 1.

**TABLE 1:** The laboratory readings of the case (pre- and post-operative).

Parameter	Pre-operative value	Post-operative value	Normal range and units
Calcium	4.42	2.01	(2.11–2.5) mmol/L
PTH	1724.56	97.13	(11–81) pg/ml
Phosphate	0.77	1.21	(0.85–1.51) mmol/L
Magnesium	0.53	0.7	(0.75–1.02) umol/L
Creatinine	165	119	(57–97) mmol/L
Vit. D	24.29	27.92	(30–100) ng/ml
ALP	250	125	(45–125) U/L
TSH	1.384	4.669	(0.56–5.91) mIU/L

PTH, parathyroid hormone; Vit. D, vitamin D; ALP, alkaline phosphatase; TSH, thyroid stimulating hormone.

### Imaging studies

**FIGURE 2:**
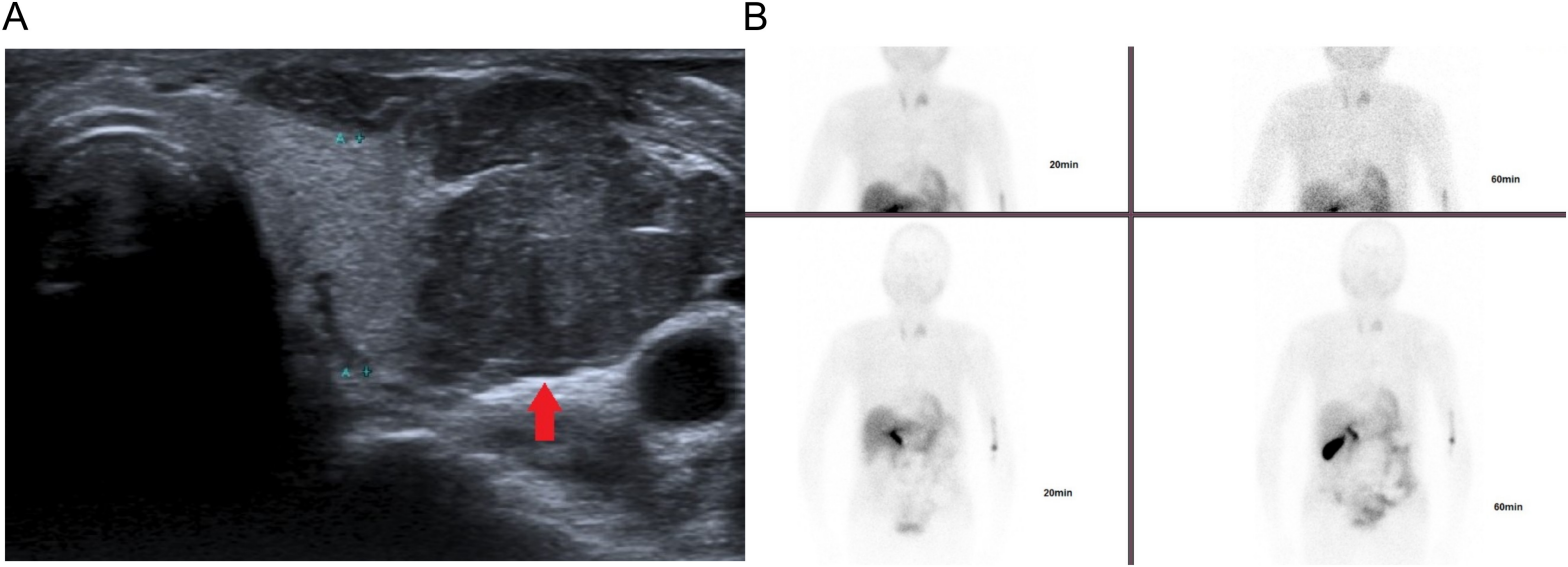
**(A)** Neck ultrasound revealed a heterogeneous hypoechoic mass (red arrow) measuring 26.6 × 20.4 mm located between the left thyroid lobe and the carotid artery. **(B)** Parathyroid 99mTc-MIBI scintigraphy: Early image (20 min): the thyroid is visualized in normal position and shape, and no obvious abnormalities are observed. The radioactive distribution is somewhat uneven. A circular radioactive uptake is seen in the middle part of the left lobe. No abnormal radioactive uptake is observed in the remaining neck or chest areas. Delayed image (60 min): in the images from different planes, the thyroid shadow appears weaker compared to the earlier image. The radioactive uptake in the middle part of the left lobe remains clearly visible.

A neck ultrasound showed a heterogeneous, hypoechoic mass (26.6 × 20.4 mm) between the left thyroid lobe and carotid artery, with increased blood flow on color Doppler flow imaging (CDFI), suggesting a parathyroid origin. No suspicious cervical lymphadenopathy was identified on preoperative ultrasound (Figure 2A).

Parathyroid 99mTc-MIBI scintigraphy: Early image (20 min): the thyroid was visualized in normal position and shape, and no obvious abnormalities were observed. The radioactive distribution was somewhat uneven. Circular radioactive uptake was observed in the middle of the left lobe. No abnormal radioactive uptake was observed in the remaining neck or chest areas. Delayed image (60 min): the thyroid shadow appears weaker in the images from different planes than in the earlier image. Radioactive uptake was visible in the middle part of the left lobe (Figure 2B).

### Surgical and pathological findings

After preoperative evaluation and obtaining consent, the patient underwent left parathyroid exploration and mass resection under general anesthesia on 23 October 2024. An irregular mass (2.5 × 3 cm) adherent to the thyroid tissue was identified in the left parathyroid region. The frozen section reported a “benign parathyroid lesion.” Post-operative pathology revealed atypical infiltrative growth with an ill-defined capsule, suggestive of parathyroid carcinoma, prompting referral to a high-level hospital for further consultation. On 7 November 2024, pathology from the tertiary hospital confirmed infiltrative tumor growth in the left parathyroid gland, extending into the adjacent muscle and thyroid tissue, and a diagnosis of parathyroid carcinoma was made. Immunohistochemistry results were as follows: PTH (+), synaptophysin (+), CD56 (+), Ki-67 8% (+), P53 wild-type.

**FIGURE 3:**
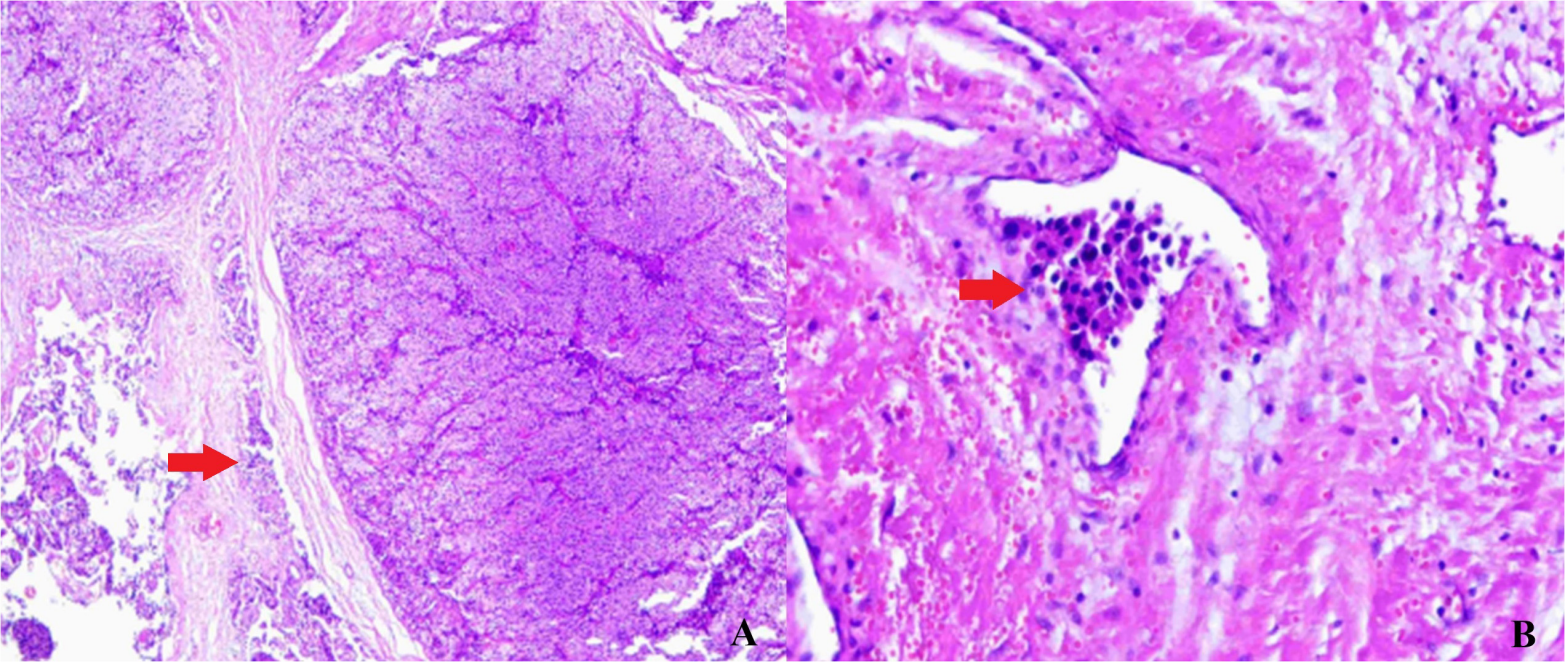
Final histopathological analysis of the case. **(A)** The red arrow demonstrates capsular invasion. **(B)** Parathyroid carcinoma with vascular invasion (red arrow) is confirmed.

Following discussions with the patient and family, radical resection of the left parathyroid carcinoma was performed on 23 December 2024. Preoperative re-evaluation revealed a parathyroid hormone level of 340.11 pg/mL and a calcium level of 2.01 mmol/L. The procedure involved resection of the left parathyroid carcinoma, left thyroid lobe, isthmus, and surrounding abnormal tissue, and central compartment lymph node dissection. Intraoperative exploration revealed no grossly enlarged lymph nodes; however, central compartment lymph node dissection was performed during the radical resection, given the final diagnosis of parathyroid carcinoma. Post-operative pathology confirmed parathyroid carcinoma with vascular invasion (Figure 3). All 17 dissected lymph nodes tested negative for metastasis (0/17). Thyroid tissue showed nodular goiter without malignant features.

### Follow-up

The patient’s mental status, appetite, and sleep were stable at discharge, with a normal body temperature. There was no hoarseness, coughing while drinking, difficulty breathing or swallowing, or numbness in the perioral or extremity areas. The surgical incision on the neck healed well with no signs of local redness, swelling, or exudate. Post-operatively, the patient was prescribed oral calcitriol, calcium supplements, and symptomatic support with regular monitoring of calcium phosphate metabolism and PTH levels. At the 6-month follow-up, there was no evidence of tumor recurrence or metastasis.

## Discussion

This case report highlights a rare and instructive instance of PC, a malignancy that presents significant diagnostic challenges owing to its clinical overlap with benign parathyroid conditions. The patient’s journey, involving three surgeries over 4 years before a final diagnosis of PC with vascular invasion, underscores the need for a thorough, multidisciplinary approach in the evaluation and management of such cases. To our knowledge, AUR as the sentinel event leading to the diagnosis of parathyroid carcinoma has been reported in only a handful of cases worldwide, underscoring the exceptional nature of this presentation. The extended diagnostic journey spanning 4 years and three surgeries underscores a critical clinical lesson: persistent hypercalcemia or recurrent hyperparathyroidism after parathyroidectomy should prompt immediate re-evaluation for malignancy, even when initial pathology suggests a benign process.

A critical reflection on the diagnostic delay reveals several missed opportunities. First, at the initial surgery, despite an elevated Ki-67 index of 8% and incomplete capsule formation, the absence of definitive capsular or vascular invasion on histopathology, combined with a benign intraoperative frozen section, led to a diagnosis of benign hyperplasia. Second, the patient’s persistent hypercalcemia and progressive mass enlargement over 4 years were not promptly investigated with repeat imaging or biopsy, in part due to the rarity of parathyroid carcinoma and the assumption of benign disease. Third, standardized Ki-67 cut-offs for parathyroid lesions were not established in routine clinical practice at the time of the first surgery. This case underscores the need for heightened clinical vigilance in patients with recurrent or persistent hyperparathyroidism, particularly when atypical features such as elevated Ki-67 or large tumor size are present.

Parathyroid carcinoma accounts for less than 1% of primary hyperparathyroidism cases and is often misdiagnosed as benign hyperplasia or adenoma. In this case, the initial surgery in 2020 suggested benign hyperplasia, despite incomplete encapsulation and focal cellular atypia. Immunohistochemistry showed a Ki-67 index of 8%; however, malignancy was not suspected. A second surgery was performed because of persistent hypercalcemia and mass enlargement, with intraoperative findings again favoring benignity. However, the final pathology revealed infiltrative growth, prompting a tertiary consultation that led to the diagnosis of PC during the third surgery. This case highlights the limitations of frozen section analysis and underscores the importance of heightened clinical suspicion for recurrent hyperparathyroidism, particularly in cases of significant hypercalcemia and large masses (5, 13). Frozen section analysis has well-recognized limitations in the intraoperative assessment of parathyroid lesions. Unlike thyroid pathology, where frozen section can reliably diagnose malignancy, parathyroid carcinoma cannot be definitively diagnosed on frozen section because capsular and vascular invasion, the definitive histopathological criteria for parathyroid carcinoma, require evaluation of the entire tumor and surrounding tissue, and cannot be reliably assessed intraoperatively. Sampling error is common, and frozen section artifacts may obscure cytological features of atypia. In our case, two consecutive intraoperative frozen sections were interpreted as benign, contributing to the decision to perform limited resections. This highlights a critical lesson: when intraoperative findings suggest malignancy (e.g., firm consistency, adherence to adjacent structures, or gross invasion), the diagnosis of malignancy should be strongly considered even if frozen section suggests benign disease, and an en bloc resection with negative margins should be considered. Emerging adjuncts, such as intraoperative near-infrared autofluorescence, may provide additional information but do not replace definitive histopathological evaluation (14).

According to the current World Health Organization (WHO) classification of parathyroid tumors, the definitive diagnosis of parathyroid carcinoma rests on the identification of capsular invasion, vascular invasion, or perineural invasion (15). Atypical parathyroid adenomas are defined by the presence of some atypical features, such as fibrous bands, trabecular growth, or increased mitotic activity, but lack definitive capsular or vascular invasion. In our patient, the final pathology confirmed vascular invasion (Figure 3B), satisfying the diagnostic criteria for parathyroid carcinoma. Notably, the initial specimens showed incomplete capsule formation and focal atypia but no definite invasion, precluding a diagnosis of carcinoma at that time. This case illustrates the diagnostic continuum between atypical adenoma and carcinoma and underscores the importance of complete surgical excision for definitive histopathological assessment.

The Ki-67 proliferation index is a potential marker for malignancy in parathyroid lesions; however, its interpretation must be considered alongside clinical and histopathological findings. Although this case showed an elevated Ki-67 index, it highlights the limitations of relying solely on this marker for the diagnosis. The Ki-67 proliferation index is a useful adjunct in distinguishing parathyroid lesions. Benign parathyroid adenomas typically exhibit a Ki-67 index below 3%, while atypical adenomas range from 3% to 5%. In contrast, parathyroid carcinomas commonly demonstrate indices between 5% and 20%. Our patient’s Ki-67 index of 8% fell within the range associated with malignancy and, in conjunction with persistent hypercalcemia and ultimately confirmed vascular invasion, supported the diagnosis of parathyroid carcinoma. However, Ki-67 alone is not diagnostic and must be integrated with clinical, biochemical, and histopathological findings (12, 16, 17). This case highlights the importance of standardized Ki-67 reporting in parathyroid specimens to enhance diagnostic accuracy and inform clinical decision-making.

The atypical presentation of AUR due to severe hypercalcemia is noteworthy. This symptom arises from multiple mechanisms, including neuromuscular dysfunction, chronic renal injury, and potential prostatic involvement. The atypical presentation of AUR due to severe hypercalcemia is noteworthy. The pathophysiology of hypercalcemia-induced AUR is multifactorial, involving neuromuscular dysfunction, chronic renal injury, and potential prostatic involvement (18, 19). At the cellular level, hypercalcemia impairs detrusor smooth muscle contractility by reducing calcium influx into smooth muscle cells, thereby diminishing intracellular calcium availability for actin-myosin cross-bridge formation (20, 21). Concurrently, elevated extracellular calcium may alter autonomic nerve conduction, disrupting the coordinated neural signals required for bladder emptying (18, 19). This results in bladder hypocontractility and functional outlet obstruction, even in the absence of mechanical prostatic occlusion (20, 21). In patients with underlying BPH, such as our patient, the combined effect of reduced detrusor contractility and increased outlet resistance creates a critical threshold for AUR. The rapid resolution of symptoms following correction of hypercalcemia, without BPH-specific surgical intervention, further supports these mechanisms. This case underscores the importance of evaluating unexplained lower urinary tract symptoms or AUR in the context of hypercalcemia, as prompt intervention can alleviate symptoms and improve patient outcomes.

In conclusion, this case offers three actionable lessons for clinical practice. First, recurrent or persistent hyperparathyroidism after parathyroidectomy warrants re-evaluation for malignancy, regardless of initial benign pathology. Second, an elevated Ki-67 index (≥5%) in a parathyroid lesion should raise suspicion for atypical or malignant behavior and prompt complete surgical excision with careful pathological assessment. Third, unexplained lower urinary tract symptoms, particularly acute urinary retention, should prompt evaluation for metabolic causes, including hypercalcemia. Recognizing this rare but clinically significant presentation can expedite diagnosis and improve outcomes in patients with underlying parathyroid pathology.

## Data Availability

The original contributions presented in this study are included in the article/supplementary materials, further inquiries can be directed to the corresponding authors.
